# Right Atrial Mass as Manifestation of Sarcoidosis

**DOI:** 10.1016/j.jaccas.2024.102695

**Published:** 2024-11-27

**Authors:** Shyla Saini, Erin Eschbach, Barry Love, Krishna Patel, Adam Jacobi, Noah Moss, Adam S. Morgenthau

**Affiliations:** a Department of Pulmonary and Critical Care Medicine, Icahn School of Medicine at Mount Sinai, New York, New York, USA; b Department of Pediatric Cardiology, Icahn School of Medicine at Mount Sinai, New York, New York, USA; c Department of Cardiology, Icahn School of Medicine at Mount Sinai, New York, New York, USA; d Department of Radiology, Icahn School of Medicine at Mount Sinai, New York, New York, USA

**Keywords:** cardiac magnetic resonance imaging, imaging, positron emission tomography

## Abstract

Cardiac involvement is relatively common in patients with sarcoidosis; however, cardiac masses are rare. Herein, we demonstrate a case of a right atrial mass with extension into the inferior vena cava, which prompted a transjugular biopsy that demonstrated non-necrotizing granulomas consistent with sarcoidosis.

## Graphical Abstract

**Figure:**
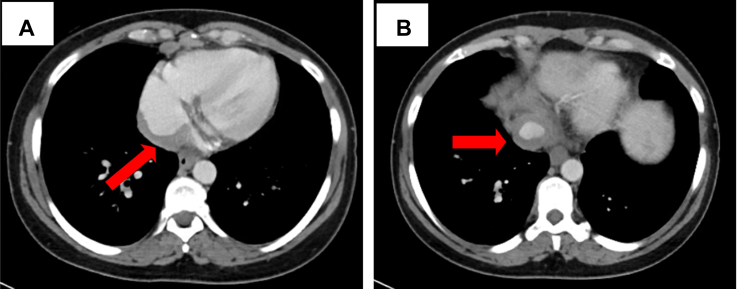

## History of Presentation

**Figure 1:**
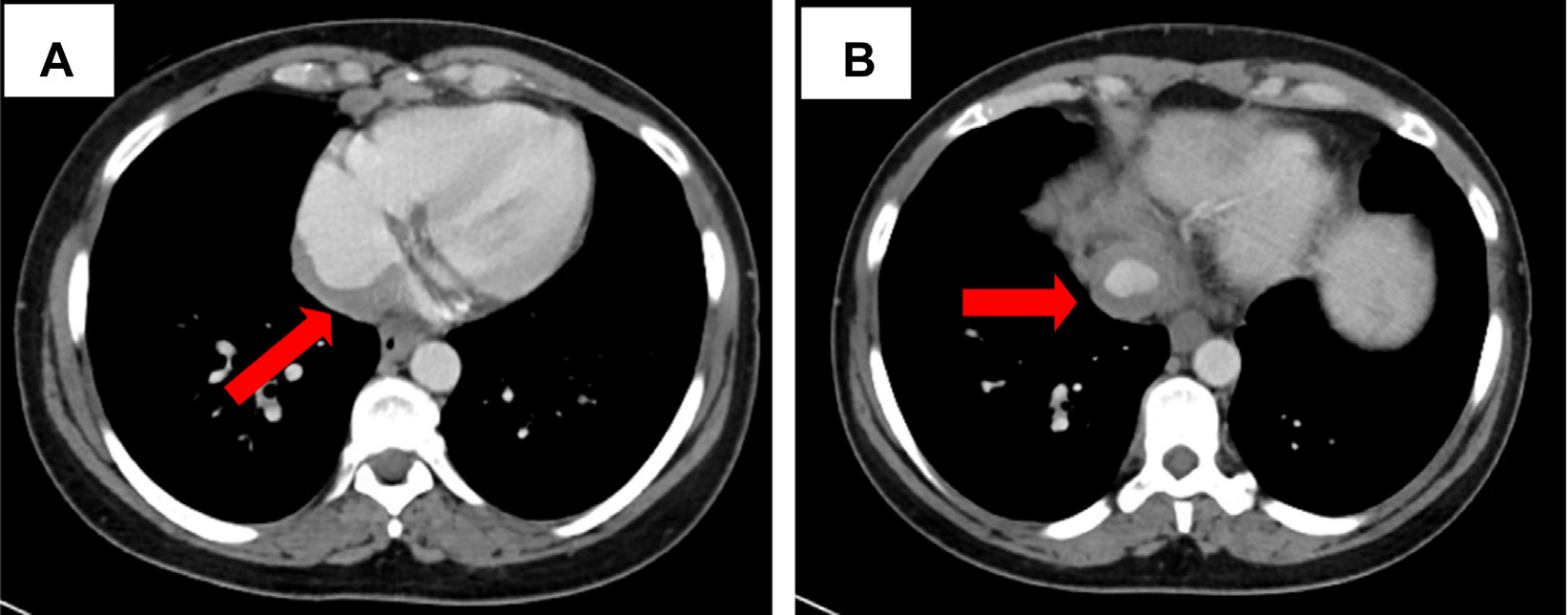
Axial Images of Computed Tomography of Abdomen and Pelvis (A) Demonstration of right atrial (RA) thickening. (B) Demonstration of circumferential thickening of inferior vena cava (IVC).

A 47-year-old African American woman presented with debilitating abdominal pain, weight loss, and night sweats during the preceding 6 months. On presentation, her temperature was 36.9 °C, blood pressure was 111/68 mm Hg, heart rate was 85 beats/min, respiratory rate was 18 breaths/min, and oxygen saturation was 99% on room air. Examination results were notable for a distended abdomen with tenderness to palpation in the right upper quadrant. Lung sounds were clear. Heart sounds were normal with regular rate and rhythm. There was no evidence of jugular venous distention or pedal edema. Computed tomography (CT) of the abdomen with contrast material (which imaged the inferior aspect of the heart) revealed a soft-tissue density in the posterior right atrium (RA) that extended into the inferior vena cava (IVC) (Figure 1).

**Learning Objectives**

• To recognize sarcoidosis as a potential cause of intracardiac mass.
• To describe the various etiologies of cardiac involvement in sarcoidosis.

## Past Medical History

Past medical history was notable for poorly controlled asthma requiring previous intubations during the preceding 10 years. In 2021, she underwent fibroidectomy, which was complicated by portal vein thrombosis (PVT).

Two months before presentation, she presented to an outside hospital for abdominal pain. CT scan of the abdomen demonstrated lymphadenopathy as well as a soft-tissue mass in the posterior RA with extension into the IVC and compression of the portal vein. An evaluation for malignancy was undertaken. CT scan of the abdomen and pelvis was unchanged compared with the previous outside study. Esophagogastroduodenoscopy and colonoscopy results were unremarkable. Core liver biopsy demonstrated focal parenchymal non-necrotizing granulomas. Fungal and acid-fast bacilli stain results were negative. Axillary lymph node fine-needle aspiration was notable for multinucleated giant cells and noncaseating granulomas. Flow cytometry of the lymph nodes and bone marrow biopsy revealed no evidence of malignancy. She was discharged on pain control, apixaban for associated PVT and provided outpatient follow-up in 2 to 4 weeks.

## Differential Diagnosis

The differential diagnosis for RA masses extending into the IVC includes malignancy (lymphoma or sarcoma), Erdheim-Chester, sarcoidosis, fibrosing mediastinitis, and immunoglobulin (Ig)G4-related disease.

## Investigations

The CT scan of the abdomen and pelvis demonstrated thickening of the posterior RA, with mass extension into the IVC. There was a Budd-Chiari appearance to the liver, consisting of infiltrative soft tissue in the periportal region, extending along the right and left portal veins and a portal vein occlusion. Blood work showed the following: aspartate aminotransferase 53 U/L, alanine transaminase 41 U/L, gamma-glutyamlytransferase 342 U/L, and alkaline phosphatase 619 mg/dL. Cardiac enzymes were normal. Antineutrophilic cytoplasmic antibody and antinuclear antibody were negative, and IgG subclasses were normal.

**Figure 2:**
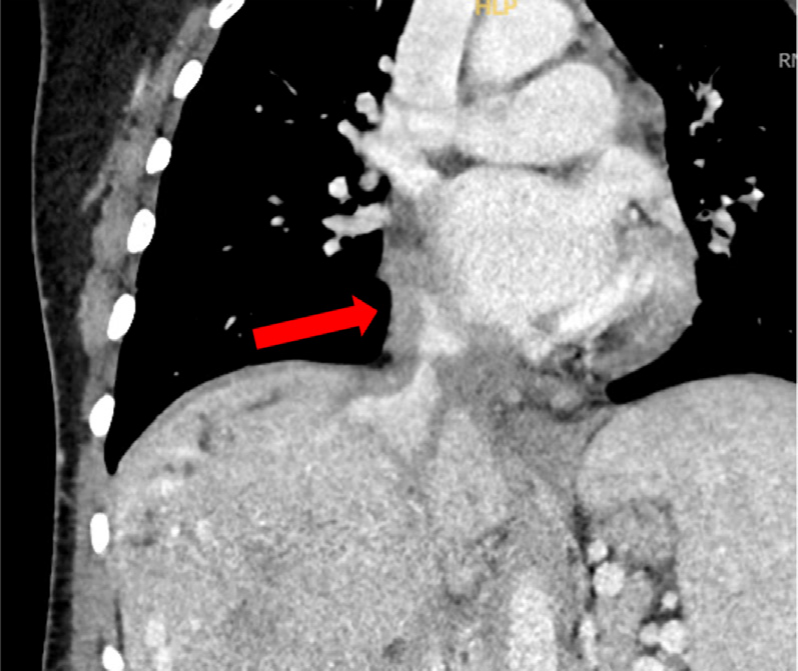
Coronal View of the Computed Tomography Angiography of Chest Infiltrative mass extending from the IVC into the RA. Abbreviations as in Figure 1.

**Figure 3:**
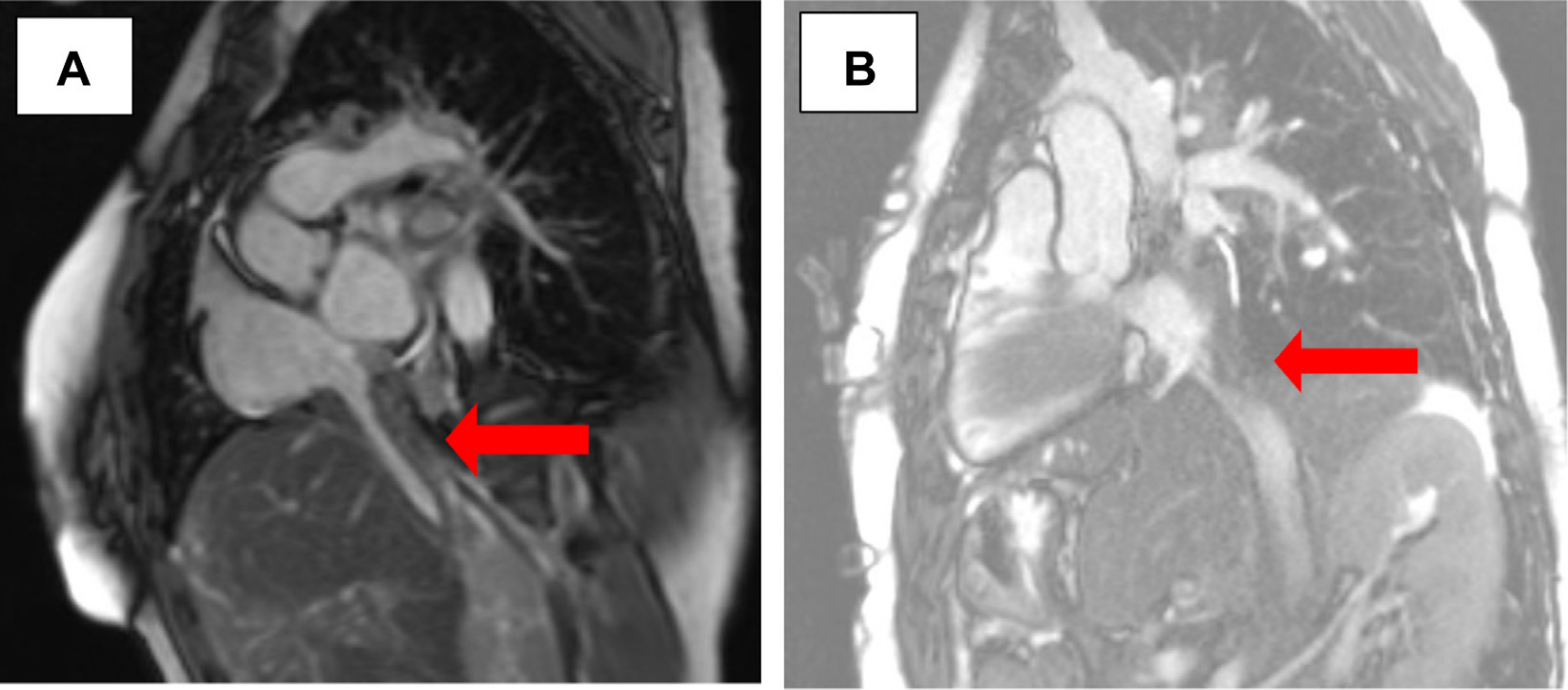
Cardiac Magnetic Resonance Imaging Eccentric wall thickening of the posterior RA with extension into the IVC, where there is circumferential wall thickening and subsequent luminal narrowing. These findings are enhanced with gadolinium. Abbreviations as in Figure 1.

CT angiogram of the chest redemonstrated the RA/IVC abnormalities (Figure 2), along with hilar and mediastinal lymphadenopathy. Cardiac magnetic resonance (CMR) imaging (Figure 3) showed eccentric wall thickening of the posterior RA, with extension into the IVC and associated luminal narrowing. On axial haste sequences, the abnormal tissue was iso-intense compared with myocardium. On early perfusion images, the abnormal tissue had faint perfusion, with evidence of diffuse late gadolinium enhancement.

A transthoracic echocardiogram did not reveal a mass even after re-review by an independent cardiologist. Bronchoscopy with endobronchial biopsies revealed multiple non-necrotizing granulomas, and transbronchial needle aspiration of the 4R and 7 lymph node stations showed non-necrotizing granulomas without evidence of malignant cells. Special stains were negative for acid-fast bacilli (AFB) and fungus. Bronchoalveolar lavage was notable for a lymphocytic predominance (54%); CD4 to CD8 ratio was 3.13.

**Figure 4:**
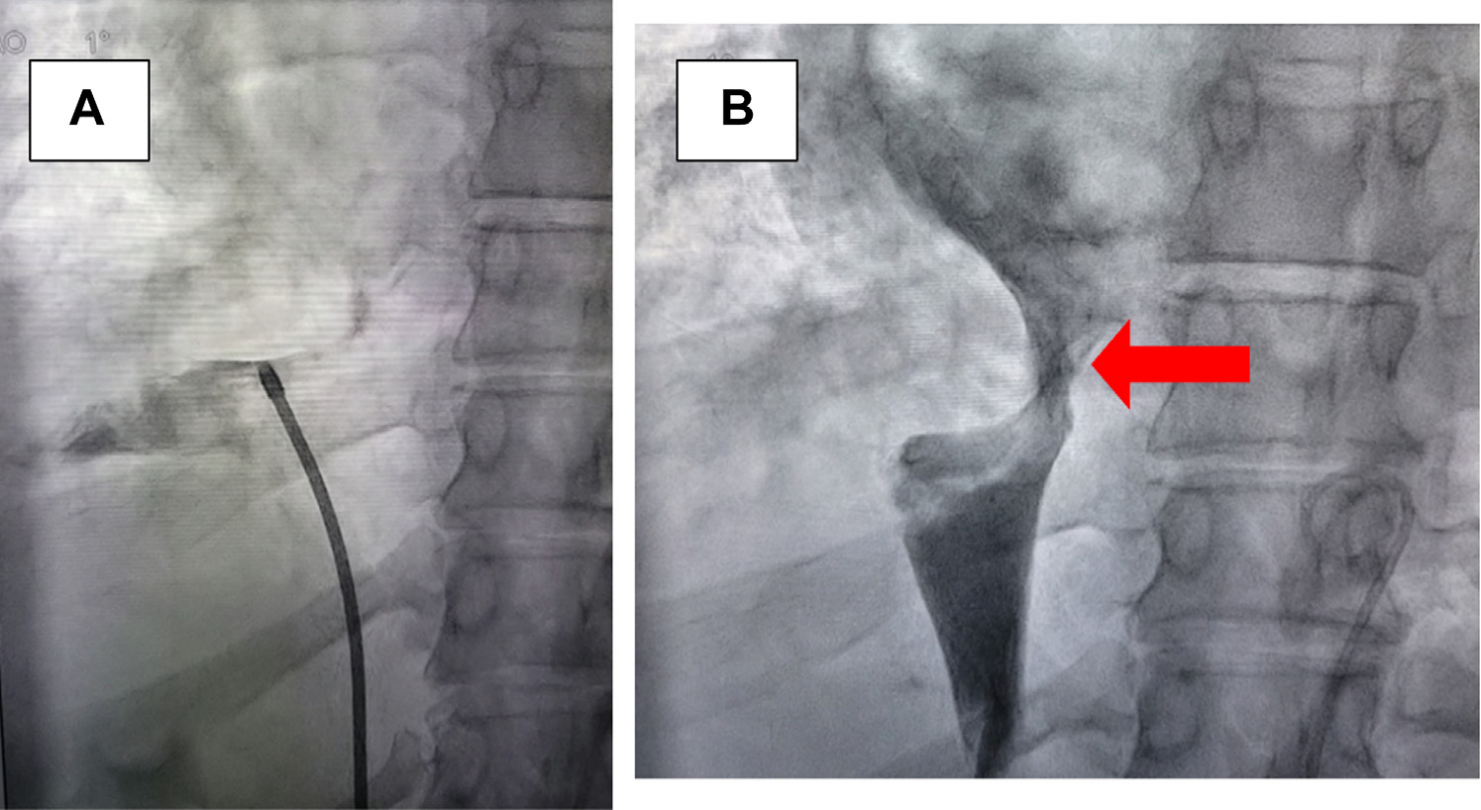
Fluoroscopic Images at RA-IVC Junction (A) Catheter seen within the IVC. (B) IVC angiogram showing marked narrowing at the RA-IVC junction, secondary to the mass. Abbreviations as in Figure 1.

Transjugular biopsy of the RA/IVC mass (Figure 4) showed multiple well-formed fibrosing epithelioid granulomas with multinucleated giant cells in portal and periportal areas. There were epithelioid granulomas noted in the wall of the vein and in a fragment of fibroadipose tissue. Portal tracts demonstrated dense fibrosis with dystrophic portal veins, and trichrome stain showed periportal fibrosis. Immunostaining showed no IgG4-positive plasma cells. According to the WASOG Organ Assessment Instrument,^1^ the diagnosis of cardiac sarcoidosis was a least “probable” but more likely “highly probable,” presuming the granulomatous infiltration identified within the IVC was similarly present within the RA.

## Management

The patient was initiated on intravenous methylprednisone (which was ultimately transitioned to oral prednisone 40 mg daily), with a plan to start infliximab as an outpatient. Her abdominal pain was managed with fentanyl patches, methadone, gabapentin, lidocaine patches, hydromorphone, and—ultimately—celiac plexus block and neurolysis for persistent pain. Surgical options were deferred given pending treatment of presumed sarcoidosis.

## Discussion

Sarcoidosis is a multisystem disease characterized by the formation of non-necrotizing granulomas in affected organs.^2^^,^^3^ Diagnosis typically involves a combination of clinical findings, pathologic evidence of non-necrotizing granulomas, and exclusion of other causes of noncaseating granulomas.^2^^,^^3^ Cardiac sarcoidosis, which may occur in as many as 30% of patients with sarcoidosis, often portends poor prognoses.^2^ Advanced cardiac imaging, including positron emission tomography (PET) and CMR, is the standard of care for diagnosis. Cardiac PET is useful in assessing response to treatment.

Cardiac sarcoidosis commonly involves the left ventricle, papillary muscles, and interventricular septum.^2^^,^^4^ RV involvement is documented in only 5.5% of cases and is associated with worse prognoses.^5^ The presence of mass lesions are uncommon. Previous case reports have described masses in the right interventricular septum,^4^ left ventricle,^6^ and pulmonary artery.^7^ To our knowledge, only 1 previous case report has documented a RA mass in a patient with sarcoidosis.^8^ In this particular case, however, biopsy of the mass was not performed. Given the uncommon nature of the RA involvement, a biopsy of our patient’s mass was performed.

Hepatic sarcoidosis is typically asymptomatic. Budd-Chiari syndrome in sarcoidosis is typically caused by external compression on the hepatic veins by hepatic granulomas and is a rare cause of congestive hepatopathy.^9^^,^^10^ The abdominal pain experienced by our patient may have been secondary to liver capsular stretch.

## Follow-Up

**Figure 5:**
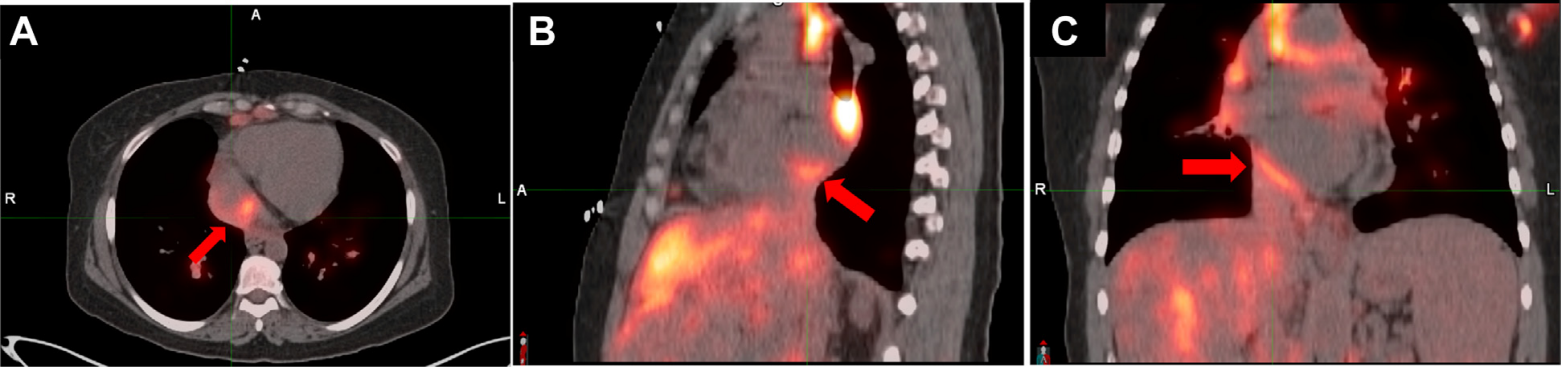
Cardiac PET Images (A) Axial, (B) sagittal, (C) coronal images of intense focal uptake in the suprarenal IVC extending into the posterior RA, suggestive of active sarcoidosis. PET = positron emission tomography; other abbreviations as in Figure 1.

PET/CMR was completed after 6 months on 40 mg of daily oral prednisone. Imaging showed persistent hypermetabolic thickening of the IVC extending to the RA (Figure 5), consistent with active sarcoidosis. There was no evidence of fluorodeoxyglucose (FDG) uptake in the left or right ventricular myocardium.

## Conclusions

Although imaging is often helpful in diagnosis of cardiac sarcoidosis, the uncommon nature of intracardiac masses warrants biopsy confirmation. Right ventricular and atrial involvement are exceedingly rare. Here, we demonstrate a case of a sarcoidosis that manifested as a RA mass extending into the IVC, which was confirmed by biopsy.

## Funding Support and Author Disclosures

The authors have reported that they have no relationships relevant to the contents of this paper to disclose.
